# Emergency department staff views of NHS 111 First: qualitative interview study in England

**DOI:** 10.1136/emermed-2022-212947

**Published:** 2023-07-06

**Authors:** Jennifer MacLellan, Joanne Turnbull, Jane Prichard, Catherine Pope

**Affiliations:** 1 Primary Healthcare Sciences, University of Oxford, Oxford, UK; 2 Faculty of Health Sciences, University of Southampton, Southampton, UK

**Keywords:** emergency department management, triage

## Abstract

**Background:**

NHS 111 is a phone and online urgent care triage and assessment system that aims to reduce UK ED demand. In 2020, 111 First was introduced to triage patients before entry to the ED and to offer direct booking for patients needing ED or urgent care into same-day arrival time slots. 111 First continues to be used post pandemic, but concerns about patient safety, delays or inequities in accessing care have been voiced. This paper examines ED and urgent care centre (UCC) staff experiences of NHS 111 First.

**Method:**

Semistructured telephone interviews were conducted with ED/UCC practitioners across England between October 2020 and July 2021 as part of a larger multimethod study examining the impact of NHS 111 online. We purposively recruited from areas with high need/demand likely to be using NHS 111 services. Interviews were transcribed verbatim and coded inductively by the primary researcher. We coded all items to capture experiences of 111 First within the full project coding tree and from this constructed two explanatory themes which were refined by the wider research team.

**Results:**

We recruited 27 participants (10 nurses, 9 doctors and 8 administrator/managers) working in ED/UCCs serving areas with high deprivation and mixed sociodemographic profiles. Participants reported local triage/streaming systems predating 111 First continued to operate so that, despite prebooked arrival slots at the ED, all attendances were funnelled into a single queue. This was described by participants as a source of frustration for staff and patients. Interviewees perceived remote algorithm-based assessments as less robust than in-person assessments which drew on more nuanced clinical expertise.

**Discussion:**

While remote preassessment of patients before they present at ED is attractive, existing triage and streaming systems based on acuity, and staff views about the superiority of clinical acumen, are likely to remain barriers to the effective use of 111 First as a demand management strategy.

**WHAT IS ALREADY KNOWN ON THIS TOPIC**

- Phone triage and online triage are offered in many countries in an effort to control ED and urgent care attendance. Some evidence suggests these services, as well as the ED itself, are more likely to be used by individuals in deprived areas.
- The 111 First initiative offered preassessment and triage using NHS 111, and prebooked appointments, to control ED attendance.
- The NHS 111 triage system has been criticised by clinicians for directing ‘inappropriate’ referrals to the ED.

**WHAT THIS STUDY ADDS**

- In this qualitative study involving staff at multiple EDs in more deprived areas of England, interviewees did not feel that NHS 111 could accurately assess the dynamic healthcare needs of ED attenders. Despite prebooked appointments, 111 First referrals joined a single triage queue in the ED to be assessed for acuity, adding to work, waiting times and frustration for all involved.

**HOW THIS STUDY MIGHT AFFECT RESEARCH, PRACTICE OR POLICY**

- The continued presence of pragmatic local streaming and preference by staff for face-to-face clinical assessment may be a continued barrier to the effective use of 111 First as a demand management strategy for NHS emergency and urgent care.

## Introduction

The seemingly unstoppable demand for emergency care services is not unique to the UK NHS; similar trends are reported globally.^1–4^ The approximately 25 million NHS ED attendances in 2019–2020 represented a 5.8% rise on the previous year following a decade or more of steep increases.^1^ While NHS ED attendances dropped dramatically in 2020–2021 during the COVID-19 lockdowns, they quickly returned to and then surpassed prepandemic levels.^5 6^

Online and telephone triage and assessment systems are a popular intervention, used in a number of countries, to manage demand and redirect patients away from the ED, but there is little definitive evidence of their effectiveness.^7 8^ In 2014, the NHS 111 telephone service was introduced to ensure that patients with urgent needs accessed ‘the right care at the right time from the right place’. In 2017, this service was also provided online. NHS 111 is available 24 hours a day, 365 days a year, offering a first point of contact for healthcare needs that ‘are less urgent than 999’ (ie, not requiring ambulance service). The telephone service is administered by call handlers, the majority of whom are not clinically trained. It uses a computerised decision support system (CDSS) to triage and assess callers, directing them to appropriate services or self-care advice where indicated. NHS 111 online uses a version of the same CDSS, designed for self-completion by patients/users, bypassing the telephone call handlers.

NHS England has suggested that NHS 111 had ‘saved over 12 million unnecessary A&E visits’.^9^ However, NHS 111 has been criticised by emergency care professionals for directing ‘inappropriate’ attenders to the ED.^10–12^ Recent research has suggested that an increase in ED attendances and ambulance dispatches would be likely if users followed the disposition recommendations offered by NHS 111 online.^13^

At the end of 2020, the NHS 111 First programme was initiated to support social distancing and control ED attendances during the COVID-19 pandemic. 111 First encouraged patients to use NHS 111 before attending the ED. Some EDs also used the NHS 111 CDSS to triage ambulatory arrivals, before admission for assessment by an ED clinician. On completion of the 111 algorithm patients identified as requiring emergency or urgent care were given a designated time slot or ‘booking’ for ED or at an urgent care centre (UCC). Similar ED ‘pre-registration’ or appointment booking systems are used in the USA, though their use has not been evaluated.^14^ 111 First was supported by national public health information campaigns, including widespread advertising advising people to ‘Think 111 First’ or to ‘Call before You Walk’. Concerns about patient safety and delays or inequities in accessing care were voiced, along with calls for evaluation.^15 16^ 111 First has continued to be used post pandemic. This paper reports our qualitative research on the experience and views of ED and UCC staff of 111 First in 2020–2021.

## Methods

Qualitative interviews were conducted between October 2020 and July 2021 as part of a larger multimethod study examining the impact of NHS 111 online on patient pathways and the workforce in England. The larger study included interviews with primary care, urgent and emergency care practitioners, and a survey of 2754 users and potential users of NHS 111 online. The part of this study presented here captured the views of ED and UCC staff about the 111 First initiative which used NHS 111 triage, including the online modality. These data enabled us to identify how 111 First functioned and to closely examine the views of healthcare professionals in urgent and emergency care about this novel system.

### Research setting

There is some evidence that use of telephone triage and assessment services is associated with socioeconomic deprivation,^17^ although this is inconsistent.^18^ Moreover, people living in the most deprived areas in England have a far higher number and rate of attendances at ED compared with other population groups.^19^ Qualitative research uses purposive sampling to select information-rich cases that can provide detailed insights.^20^ In order to collect rich data from areas with high need/demand, most likely to be using NHS 111 services (including 111 First), we purposively recruited interviewees from areas in England with high deprivation scores and mixed sociodemographic profiles in terms of age and ethnicity. For the part of the study reported here, this comprised nine sites in the Midlands, East, South and South East of England, that included regional trauma centres, local and district general hospital EDs, minor injury units and UCCs.

### Participants

We interviewed 27 staff working in these urgent and emergency care services (table 1). These included service commissioners, managers, clinicians (different grades of nurses and doctors) and administrative staff. They were invited to take part through their service lead by email, provided with an information leaflet and asked to provide verbal consent. Interviews took place over the telephone at a time convenient to the participant. We interviewed between two and seven staff per site, except for one interview (ED10) who was the sole interviewee from major trauma centre 2.

**Table 1:** Interview participant roles and site

ID	Site	Role
ED01	Major trauma centre 1, East England	ED nurse
ED02	Major trauma centre 1, East England	Receptionist
ED03	Major trauma centre 1, East England	Pharmacist
ED04	Major trauma centre 1, East England	ED nurse
ED05	Major trauma centre 1, East England	ED doctor
ED06	Major trauma centre 1, East England	Senior manager
ED07	Local trauma unit 1, South East England	ED doctor
ED08	Local trauma unit 1, South East England	ED Nurse
ED09	Local trauma unit 1, South East England	ED doctor
ED10	Major trauma centre 2, South England	Senior manager
ED11	Major trauma centre 1, East England	Senior manager
ED12	Local trauma unit 2, East England	ED nurse
ED13	Local trauma unit 2, East England	ED doctor
ED14	Local trauma unit 2, East England	ED doctor
ED15	Local trauma unit 2, East England	ED doctor
ED16	Local trauma unit 2, East England	ED doctor
ED17	Local trauma unit 2, East England	ED nurse
ED18	Local trauma unit 3, South East England	ED nurse
ED19	Local trauma unit 3, South East England	ED nurse
ED20	Local trauma unit 3, South East England	Receptionist
ED21	UCC 1, Midlands	UCC nurse
ED22	UCC 1, Midlands	UCC nurse
ED23	Local trauma unit 4, South East England	ED doctor (trainee)
ED24	Local trauma unit 4, South East England	ED doctor (trainee)
ED25	UCC 2, Midlands	Receptionist
ED26	UCC 2, Midlands	Receptionist
ED27	UCC 2, Midlands	UCC nurse

UCC, urgent care centre.

### Interviews

An interview topic guide was developed and piloted by JT and CP to explore the impact of NHS 111 on the workload and arrangements of urgent and emergency care services (online supplemental file 1). Data were collected through single-episode one-to-one interviews between October 2020 and July 2021 conducted virtually online or by telephone by experienced qualitative researchers (JM, JT and CP). All three interviewers are women: one (JM) is clinically trained as a nurse, and JT and CP have together accumulated over 45 years’ experience using mixed methods to study health services and access systems. Recruitment continued until the research team agreed that data saturation had been reached. Interviews lasted between 15 min and 50 min with an average of 24 min. (The sole 15 min interview was with a participant who said they had never heard of NHS 111 online or 111 First.) Interviews were audio recorded, transcribed and loaded into NVivo V.12 to support coding and retrieval.

10.1136/emermed-2022-212947.supp1 Supplementary data

### Analysis

We analysed the data thematically, drawing on the approach detailed by Braun and Clarke.^21^ The primary researcher (JM) coded the deidentified transcripts inductively, then used a draft coding framework developed and revised in consultation with the wider research team (JT, JP and CP). Themes were identified and refined by grouping related codes together and exploring comparisons using matrices/charts and mind maps to facilitate the analysis. The ED/UCC interviewees provided insights about the (not inconsiderable) work associated with the deployment of NHS 111 and the 111 First initiative. We developed a high-level code to capture views and experiences of 111 First within the full project coding tree, and from this, were able to construct two explanatory themes focused on these data. Throughout the process, interviewer notes and team discussions were used to examine and question assumptions and judgements to support reflexivity and trustworthiness of analysis.

### Public and patient involvement

PPI contributors, recruited through support organisations, included two digitally confident members, six people identifying as differently abled, five people living in an area of urban deprivation with a multimorbid population, and five care navigators working with people experiencing homeless and substance misuse. All participants had experience of NHS 111 and attending ED. The research team were in regular contact with members throughout the study. To challenge assumptions and augment the analysis, PPI members were asked to comment on and consider the veracity and credibility of themes and interpretations.

## Results

Two themes relating to the experiences of ED/urgent care practitioners of 111 First were identified.

### First was additional to local streaming practices

EDs had pre-existing local triage and assessment systems to prioritise attenders. A triage or ‘streaming’ nurse was typically deployed to see all patients relatively soon after initial registration with reception. These local systems were used for all ambulatory arrivals, whether ‘booked’ via 111 First or those who had not used NHS 111:

The streaming nurse who sits at the front desk will do the same job if they are referred, not referred, have an appointment or not. They have to still queue, they have to still wait to be seen by the same person. (ED17, ED nurse)

A few participants described 111 First as helpful, either in directing patients away from ED to the most appropriate provider or in warning staff what conditions were coming in. However, several reported that this external booking process and allocation of an arrival time were a source of confusion for patients.

111 were able to book you an appointment, so to speak, in A&E. [implying that] if you turned up between these times you would get seen and I think that caused a bit of confusion with the patients as well because I think they thought they were coming to see a doctor at 10 o’clock for example and that they would be seen straight away and that wasn’t the case. (ED18, ED nurse).

ED reception staff reported that 111 First ‘bookings’ created an expectation that patients would be seen immediately. This was often not possible due to the acuity of other arrivals at the ED.

Patients are saying, ‘we were given an appointment and told we would be seen at this time’ and we have to clarify to them that this is how we are working and you have to wait for your turn to be seen. (ED23, ED doctor (trainee))

The presence of local streaming systems meant that patients who understood that they had been booked into the ED by the NHS 111 phone service could also be redirected away from the ED on arrival. Streaming sometimes managed demand by diverting cases to general practitioners (GPs) colocated within the ED, or as in the following quote, by booking a GP appointment with the patient’s own GP:

they say they called the GP and there is no appointment and we will dial the GP number and we will get the appointment for them this afternoon. So go to your GP. (ED13, ED doctor)

In some sites, ED attenders were asked to complete the 111 online algorithm before gaining entry to the department. Some patients received the disposition of ‘speak to your GP’ and were turned away at the ED. However, faced with such patients ‘on the doorstep’ staff might over-rule the 111 disposition and send them in to the ED anyway:

We still see a depressing number of calls when the patient is in the ED and their notes in the system say ‘the patient is in ED so I booked them an appointment’. (ED10, senior manager)

111 First was seen as an additional triage system and crucially one that could not substitute for local streaming systems and triage practices. While it appeared to offer a fast-track or prebooked appointment, the filtering of all attendances into a single queue on arrival undermined this. The resulting unanticipated waiting was described by participants as a source of frustration for staff and patients alike:

that’s probably the frustration between the hospital, GP and the people in the general public. We don’t all have the same idea of what it is trying to achieve and it can be manipulated when it is pushed [busy]. (ED18, ED nurse)

### Triage was not seen as a substitute for clinical acumen

Participants expressed concern about remote telephone and online triage and assessment. They described how their own clinical decision making was guided by context specific, complex, flexible and interpretive knowledge. They felt this was best gathered directly from the patient (ideally by a clinician) in a face-to-face consultation:

It is because the biggest flaw of 111 is you are never going to provide decent healthcare unless you can sit the patient in front of you, put your hands on and see what is going on. (ED18, ED nurse)

the 111 service is actually quite crude to certainly a GP at the end of the phone, any health care professional, it’s that clinical acumen, listening to that patient and actually hearing what is going on and no fancy computer system will be able to replicate that. (ED09, ED doctor)

Participants did not feel that NHS 111 could accurately assess dynamic healthcare needs and were not convinced that telephone or online assessments were adequate. Some participants were explicit that they trusted their clinical skills/acumen more than the 111 algorithm.

I will always discuss with the patient why they are there and see if it matches up. I would never just trust that 111 paperwork. (ED19, ED nurse)

I understand that if you are making telephone or online assessments you have multiple factors that make that assessment much more difficult. (ED27, UCC nurse)

One participant recounted an interaction with an ED consultant which highlighted the view that streaming was not only about diverting patients away from the ED, it was about making ‘safe decisions’. However, though participants acknowledged the NHS 111 algorithm must chose the safest option, it was viewed as a source of ‘inappropriate referrals’ by some.

There has been a few inappropriate ones [referrals to ED] but it’s not easy to make that [judgement] by 111 staff who are not maybe fully clinical or fully trained. …The algorithm will want to make sure it’s the safest option, you know if someone rings with chest pain they need to see cardiac and if someone has abdominal pain they want to alert acute abdomen. It’s challenging, the algorithm. (ED14, ED doctor)

my experience of 111 through patients and not for myself is not necessarily particularly good. I’m seeing awfully large numbers of patients coming through 111 who shouldn’t be there or should go to the GP (ED24, ED doctor)

Clinical acumen was prized and respected; by contrast, the NHS 111 algorithm used for 111 First was not. Distrust of NHS 111 assessments meant that the algorithm and the 111 First initiative were perceived as a source of ‘inappropriate attendances’ resulting in high ED workloads and long waiting times. NHS 111 was viewed as inferior to the clinical assessment that staff could provide in person. These views reinforced the continued use of local streaming practices and fuelled negative views about the 111 First initiative.

## Discussion

The deployment of remote assessment to support triage and streaming of patients before they present at ED is attractive in the face of rising demand and is being used in different countries. Commercial triage and prebooking tools have been developed. For example, InQuicker^22^ is used in the USA and eTriage^23^ is used in some UK EDs. The NHS First initiative was, to our knowledge, the first use of a non-commercial triage, assessment and ED prebooking system in a national health service. While a 2021 survey of UK patients judged that 111 First was ‘useful to patients when it works well’, this survey, like our data, identified frustration by people who had used the system.^24^ Our data show that 111 First did not substitute for local streaming and triage practices that assess the acuity of all patients who attend. NHS 111 was an additional assessment point and, thus, delay in patient journeys to care. Patients booked by 111 First arrived at the ED or UCC to discover that they needed to be assessed again alongside other attenders before joining a new queue to be seen/treated. This was reported by participants as a source of frustration and difficulty for staff and patients: the ‘extra queue’ led to anger and (sometimes) abuse of reception and frontline staff from patients, and/or increased patient distress.

The finding that frontline staff favour clinical acumen is not unique to this study; a BMJ opinion piece in 2021 expressed concerns about the risks when ‘triage does not happen face to face’.^25^ Staff working in EDs and UCCs valued in-person clinical triage and assessment, viewed the NHS 111 algorithm as inferior and associated it with inappropriate attendances. The suggestion that NHS 111 encourages unnecessary use of emergency care services has been made elsewhere.^26 27^ In a 2021 survey^28^ of members of the UK Royal College of Emergency Medicine, over 50.7% of people responded that NHS 111 and phone first services ‘had increased or significantly increased demand in their ED, while only 3.6% responded that it had decreased or significantly decreased demand, showing that this service is currently not fulfilling its purpose’. Even if the views identified in our analysis are not supported by evidence, they feed negative perceptions and responses to 111 First.

### Limitations

Interviews were conducted during the COVID-19 pandemic in the UK when pressure on ED and healthcare staff was high. As with other qualitative interview studies, our work provides a snapshot of views and experiences. Our purposive sampling meant that we were able to include participants from a range of urgent and emergency care settings, and we deliberately included settings characterised by deprivation and high health need, which could be expected to benefit most from the use of 111 First. While these design features mean that these data and analyses cannot generalise to all settings, the use of local streaming practices and staff views about in-person assessment are unique neither to the pandemic setting nor to our study sites.^10 11^ We are confident that our findings remain applicable to the postpandemic context and other UK ED/UCC settings.

### Implications

The continued increase in ED attendances is impacting all sections of the health service.^29 30^ The appropriate use of emergency care is a complex and multidimensional problem that requires context specific, targeted and integrated interventions.^7^ Inability to get an appointment with a GP has been linked to a rise in local ED attendance.^31^ One study has suggested that increasing access in primary care can reduce paediatric attendance at the ED,^32^ and multidisciplinary case management may reduce ED visits for frequent attenders.^7^ However, difficulties in securing timely GP appointments, concerns about the accuracy of diagnosis in primary care, and perception that diagnostic tests are done more quickly in ED and/or that ED doctors are more knowledgeable^33^ all continue to drive demand for ED care. The continuation of remote access to primary care following the pandemic may also have contributed to an increase in the presentation of non-acute symptoms to ED by some patients. This has been linked to perceptions that remote consultations lead to less accurate diagnosis because they cannot provide visual and tactile information about presenting complaints. There are also concerns that digital exclusion, for example, by older people, people not confident in English and people on low incomes, may drive ED attendances by these groups.^6^ In December 2022, the UK’s British Medical Association’s England GP Committee issued guidance for the assessment of urgent problems which stated that patients who can wait should be placed on a waiting list if safe capacity for appointments has been reached for the day.^34^ Such attempts to manage demand in primary care, which increase waiting for primary care, may encourage patients to seek help from local EDs which are seen as providing quicker access to care.

The policy vision of joined-up digital care continues to drive refinement and expansion of the NHS 111 service.^35 36^ However, the use of NHS 111 for preassessment adds a further layer of triage, in addition to local streaming, and is a source of frustration for staff and patients and introduces further delay in accessing care for patients. Our interviewees did not perceive that 111 First was successfully redirecting patients to primary care, especially as many patients continue to seek (and prefer) in-person care, which can be accessed 24 hours a day in ED if primary care is unable to respond.^37 38^ Our study describes important views and experiences of frontline staff in EDs and UCCs that may prove to be significant barriers to the effective operation of 111 First to manage ED demand.

## Conclusion

111 First was introduced during the COVID-19 pandemic to manage in person ED attendances and continues to be promoted. However, ED acuity-based triage and streaming systems and staff views about the superiority of clinical acumen and face-to-face triage resulted in a second queue for people triaged by NHS 111, creating duplication and delays that have been a source of frustration for both patients and staff. These factors may prove to be a continued barrier to the effective use of 111 First as a demand management strategy for emergency and urgent care.

## Data Availability

Data are available upon reasonable request.
