# Supplementary material for: Emergency department staff views of NHS 111 First: qualitative interview study in England
Source: Emerg Med J. 2023 Jul 6;40(9):636–40. doi: 10.1136/emermed-2022-212947 (PMC10447374; doi:10.1136/emermed-2022-212947)
Supplement: Supplementary data [file emermed-2022-212947supp001.pdf]

UNIVERSITY OF OXFORD

Ethnographic study of patient pathways and workforce implications of NHS 111 Online

NHS 111 Online: Case study interview topic guide

Provide info leaflet and consent form as required. Talk through and answer questions.

Preamble: We are interested in the impact - if any – the new NHS111 online services on your work/ what you do here in [setting], but perhaps you could begin by telling me a little bit about your role here – what is your work role? What services are provided here?

What is your understanding/knowledge of NHS 111 online? *[Probes: what is it for, how does it work, if necessary compare to telephone version, what do you think about the service, any experience of use?]*

Are you aware of receiving /seeing people here at [site] who have used NHS 111 online before they arrive here? *[Probes: what kinds of cases - same/different to 'usual' attenders]*

> if yes - What impact has this had on your work/ what you do here in [setting] – if any, *Probes: impacts on workforce, skills required, time and other resources?*
>
> Has this changed over time? How (Increase/decrease) ?
>
> Are the impacts felt differently on different days/times?

What do you think are the longer term implications of NHS 111 online / where do you see services heading? [*Probes: what are the broader benefits/challenges of the system?*]

Is there anything else about NHS 111 you want to mention?

NHS 111 Online Case Study Interview Topic Guide V1.0 30.01.2020
Study title: NHS 111 Online Study.
IRAS project number: 272729
Chief Investigator: Catherine Pope
REC Reference Number: 20/LO/0294
